# Impaired Initiation But Not Execution of Contralesional Saccades in Hemispatial Neglect

**DOI:** 10.1155/2002/253201

**Published:** 2002-08-01

**Authors:** Marlene Behrmann, Thea Ghiselli-Crippa, Ilaria Dimatteo

**Affiliations:** ^1^ Department of Psychology Carnegie Mellon University PA USA; ^2^ Department of Neuroscience University of Pittsburgh PA USA; ^3^ Department of Information Science University of Pittsburgh PA USA; ^4^ Department of Statistics Carnegie Mellon University PA USA

**Keywords:** eye movements, saccades, hemispatial neglect, parietal cortex

## Abstract

Patients with unilateral neglect are impaired at making saccades to contralesional targets. Whether this problem arises from a deficit in perception, in planning the saccade or in executing the eye movement or some combination thereof remains unclear. We measured several variables related to the initiation and execution of saccades in an experiment which crossed two factors: target side (left, right) and direction of saccade (leftwards, rightwards). Relative to control subjects, patients with left-sided neglect were impaired in planning but not executing the contralesional saccade; while the latency to move their eyes following the onset of the target was increased, the duration and velocity to reach the target were normal. In addition, there were also no directional differences for saccades that were hypometric or inaccurate in the patients, further ruling out an execution impairment. Interestingly, this directional initiation deficit was exaggerated for leftward saccades to left targets, compared with all other conditions. We suggest that the disadvantage for contralesional saccades in neglect patients is attributable to a deficit not only in perceiving contralateral targets but also in planning leftward saccades. Once the saccade is initiated, however, execution apparently proceeds unimpaired.

## 1. Introduction

In order to move one's eyes to fixate a desired target, one needs both to perceive the target and locate its position as well as to execute a saccade towards the selected target. Patients with hemispatial neglect following a lesion to parietal cortex are known to acquire oculomotor targets poorly. Despite the absence of detectable fundamental oculomotor deficits, such as gaze paralysis or optic ataxia, these patients show a direction-specific deficit of saccadic orienting [6,9,30, 43,44,50]. For example, they make fewer contralesional than ipsilesional saccades, are slower to initiate leftward saccades, make multiple small saccades to locate the contralateral target, have prolonged search times for ipsilesional targets and adopt an ipsilesional (rather than contralesional) position for starting their oculomotor visual exploration [19,20,30,31,40,41,73, 82,84]. Although these behavioral phenomena have now been described in detail, it remains unclear what gives rise to the impairment in eye movement.

One possible explanation for the observed contralesional deficit is that it arises solely from the failure to perceive the target. Thus, the difficulty in representing the target and its position or, perhaps disengaging from the current spatial position [67], leads to a delay in initiating a required saccade. Alternatively, the problem could arise in planning the action itself, independent of a perceptual or attentional problem. A final possibility is that the deficit might manifest in the actual execution of a saccade. Of course, these are not mutually exclusive possibilities and more than one underlying deficit could contribute to the impairment. The goal of this paper is to explore in detail the mechanisms giving rise

ISSN 0953-4180/01/02/$8.00 © 2001/2002 – IOS Press. All rights reserved to the eye movement pattern in a group of patients with hemispatial neglect. We first describe the neurobehavioral disorder and then review the evidence favoring an impairment in the planning of action in hemispatial neglect. Finally, we outline those findings that argue for a disorder that also affects the execution of action in these patients. Because there are only a few studies on eye movements that clearly distinguish between the initiation and execution of action, we appeal to the relevant literature from reaching studies conducted with this same patient population.

## 2. Hemispatial neglect and eye movements

Hemispatial neglect is a neuropsychological disorder in which patients fail to orient to or process information from the side of space opposite the lesion. The deficit is more frequent after right than left hemisphere lesions (hence, we refer to neglect as left-sided throughout this paper) and the inferior parietal cortex is the most frequently affected neuroanatomical region [13,59,46,81]. The behavioral consequences of such a lesion may be far-reaching and may manifest in the patients' drawing and copying, in their personal care and in multiple sensory modalities, including vision, audition, olfaction and somatosensation. Importantly, the impaired response to contralesional versus ipsilesional stimuli is not attributable to a primary sensory or motor disorder. Instead, neglect is generally interpreted as a failure to represent contralateral spatial information [13,18,26, 34]; when cued to attend or orient to this side, these patients are able to do so, although, left to their own devices, they usually do not [51,72].

In addition to the behaviors described above, neglect may also involve a directional bias in motor control, which disadvantages movements towards contralesional stimuli. This contralesional disadvantage manifests in two different ways. The first, termed 'directional hypokinesia' refers to a deficit in planning an action, according to the original definition provided by Heilman and colleagues [38], although this term is sometimes used more broadly than this definition. This initiation problem contrasts with 'directional bradykinesia' which also gives rise to contralesional slowing but, in this case, the problem is in the execution of the movement. Directional bradykinesia may manifest in slowed duration but might also be revealed in altered velocity or amplitude of the movement or even in a circuitous trajectory.

To the extent that studies of the eye movements of patients with hemispatial neglect have been conducted, they have focussed almost exclusively on the difference between the contralesional and ipsilesional sides in the number of saccades, the duration of fixations or the time to initiate a saccade, as measured from the onset of a specific target (for example [10,37,47]). The overwhelming finding from these studies is that these patients are disproportionately impaired, relative to their control counterparts, on all these measures. Although there is consistency in showing that these patients have a directional hypokinesia in the oculomotor domain, very little emphasis has been placed on evaluating the execution or bradykinesia of the eye movements. One recent exception to this is the study by Niemeier and Karnath [62] in which they had neglect patients and control subjects perform a visual search task either for a laser spot or for a nonexistent target letter in complete darkness. Of interest is that the patients showed a reduction in saccadic amplitude, relative to the controls. However, this amplitude reduction was apparent in all directions, not just contralesionally. Based on these findings, these authors argue that there is no deficit in executing saccades contralesionally in hemispatial neglect (but see [82] for a different result with no apparent deficit in saccadic amplitude at all). The primary finding of an impairment in initiation and not in execution is also consistent with data from a recent study with nonhuman primates. Following a muscimol injection in various sites in LIP [53], only the latency of saccades was increased, but neither the velocity nor the duration were adversely affected. Taken together, then, the results suggest no alteration in the metrics or dynamics of the execution of saccades following parietal lesions but, instead, point to a clear problem in their initiation.

## 3. Hemispatial neglect and manual movements

Despite the fact that the eye movement deficit appears to be manifest in the planning rather than in the execution of the action per se, data from reaching studies with neglect patients have pointed out deficits in both processes. For example, several studies have found that patients with neglect were both slower to initiate as well as to execute contralesional manual movements, and this was exacerbated by the presence of a visual distractor [11,39,55,56]. Among the execution problems documented are alterations of peak velocity of the contralesional movements, prolonged acceleration time, and more epochs of acceleration and deceleration, relative to the control subjects [58]. Importantly, the presence of the deficit depended on the lesion site: whereas patients with frontal lesions showed both the hypokinesia and bradykinesia, parietal patients showed only the hypokinesia [11,39,55].

In addition to changes in the temporal parameters of the action, some studies have also reported spatial problems such as a systematic distortion of manual movements towards the ipsilesional side [32]. This distortion is exacerbated in the absence of visual feedback [35] and in the presence of distractors [16,21]. However, not all studies agree on the presence of directional bradykinesia. For example, Karnath and colleagues have shown no direction-specific motor impairment in the reaching performance of neglect patients on either spatial or temporal measures [45,49]. Although there were some alterations in the patients' performance, these were either seen for both ipsilateral as well as contralateral movements or were also seen in the non-neglect control group, suggesting that these alterations are not directly associated with hemispatial neglect. Based on these results, the authors argue against a direction-specific bradykinesia in neglect although they recognize that a generalized bradykinesia may exist.

How can we reconcile the discrepant results reported above? An important consideration concerns the starting position of the hand. In the Konczak and Karnath [49], experiment, all movements originated in the right hemispace and were performed to targets in the right and left hemispace. In contrast, Mattingley et al. [58] found evidence of directional bradykinesia only when rightward movements originating in left hemispace were compared with leftward movements with a starting point in the right hemispace. Indeed, previous research has also shown that the starting position of the hand may influence the performance of neglect patients [27]. The issue of starting position of the hand has been taken up with great care recently by Mattingley and colleagues [39,57]. In their studies, patients with frontal or with parietal lesions reached to targets in the left or right field starting from a position located at the body midline or located to the left or right of their body (and of the target). This paradigm allowed them to answer two questions: the first concerned the differential pattern which might occur between patients with frontal and patients with parietal lesions. As noted previously, the deficit in execution appears to be specific to the former group whereas both groups show a problem in action initiation. The second, more relevant question for our purposes, concerns the influence of starting position of the hand. In this paradigm, sensory information about the target remains constant and direction of movement is manipulated (for example, left target, left reach; left target, right reach). Patients with parietal lesions showed significantly slower reaction times to left than right targets, as measured from the onset of the target to release of the start button. This pattern, however, was modulated by the start position of the hand. When the left target was acquired by a leftward reach (start position center), performance was significantly worse than when the left target was acquired by a rightward reach (start position extreme left) (see [42] for additional evidence on abnormal reaches to visual but not proprioceptive targets). The same was not true in frontal patients who showed slowed left target reaches but who were unaffected by hand starting position. The critical result for the current paper is that patients with parietal lesions showed an initiation deficit for left targets. Although this deficit may have a perceptual basis (left targets always slower than right targets), there is also a motor planning component in that leftward reaches are more slowly initiated than rightward reaches to the same target.

## 4. Relationship between oculomotor and manual movements

As is evident from the above review, the reaching studies are more advanced than the corresponding eye movement studies. At present, we do not know much about the execution of saccades by parietal patients nor do we know whether there is a modulation of target acquisition by the starting point of the eyes. If the same mechanisms mediate both eye and hand movements, then we might expect to see similar results in the oculomotor and manual motor domains. However, whether or not the same mechanisms mediate the planning and execution of eye and hand movements remains controversial. Behavioral studies with normal subjects suggest that the oculomotor and manual motor systems are closely interrelated during the production of an aimed movement and therefore predict similar findings with eye and hand movements in neglect patients. Data supporting this correspondence between response types come from a study by Abrams, Meyer and Kornblum [1] who found that people almost always direct their eyes to a target to which a manual movement is made, and that manual movements are less accurate if a subject is prevented from moving the eyes. Perhaps more dramatically, Bekkering et al. [12] found that saccadic adaptation transfers to the manual motor system; pointing movements made by an unseen hand were shorter when the eyes were adapted to make short movements than when their eyes were not adapted, suggesting that, at some level, they rely on a common code or signal. Other behavioral evidence, however, reveals differences between the systems. In a paradigm in which subjects executed manual or eye movements and an occasional stop signal was presented, reaction times to inhibit the signal were faster for the eye than for the hand movements [54].

The neurophysiological data concerning the equivalence of hand and eye movements is also somewhat unresolved. For example, despite the anatomical segregation of parietal cortex into two regions, one associated with eye and the other with reach movements [7], both movement types share a reference frame that is defined with respect to eye-centered coordinates [69, 79]. Other, conflicting findings, however, argue for distinctions between the systems: whereas oculomotor centers represent space in an oculocentric reference frame [3,22,23], neural activity in motor or premotor cortices prior to an arm movement codes the direction of reaching movements, independent of amplitude, in a body-centered reference frame [33], supporting both structural and functional segregation of the two systems.

But most directly relevant for us is a recent neuropsychological investigation in which the eye and hand movements of neglect patients appear to be governed by separate systems [52]. In this study, patients responded to targets either manually, ocularly or, in both ways simultaneously. If manual and oculomotor responses arise from the same fundamental system, behavior should be correlated across response type. This, however, was not so. When patients responded manually, they showed the typical attentional gradient associated with neglect, with faster responses to the more rightward target in both fields. When they responded manually but with accompanying eye movements, the responses obeyed the retinal eccentricity effect, notably, faster responses to targets closer to the fovea, in both visual fields. Because the different responses adhere to different psychophysical functions, the authors [52] concluded that the parietal lobe does not play a role in oculomotor programming per se and that the saccadic deficit is purely a consequence of a general disruption of a visual spatial map and/or the ability to remap space to account for changes in eye position [29, 36].

Given the controversy in the reaching literature as well as the questionable correspondence between the oculomotor and manual systems, in the present study, we examine the initiation and execution of eye movements in a group of neglect patients to address two main issues. The first issue is whether the directional deficit in oculomotor behavior manifests in the poor perception of targets, in the planning of the action and/or in the execution of the saccade. The second question we address is whether the directional bias against left- versus right-sided targets is influenced by the starting position of the eyes. To examine whether any observed directional bias for targets on the left is exaggerated for leftward saccades, we adapted the methodology of Mattingley et al. [57] to the oculomotor domain by altering the start positions of the eyes while measuring the planning and execution of saccades (see also [39]). In this experiment, subjects were seated in an arc of LEDs and were required to saccade toward an illuminated LED which could appear randomly to the left or right at one of several possible locations. The eyes could either be centered on the midline of the LED array, along with the head and trunk, or could start from the extreme left or right. We first present the data from the eyes-center condition in order to explore the presence of an initiation and/or execution deficit. Thereafter, we include the data from the different fixation conditions to examine the contribution of saccadic direction to the directional-specific eye movement deficit.

## 5. Methods

### *5.1. Subjects*

Two subject groups consented to participate in these experiments, a group of normal control subjects, and a group of patients with hemispatial neglect. All subjects were right-handed and English-speaking and had normal or corrected to normal visual acuity (see below). No subject had cataracts, glaucoma or optic neuropathy and no subject had amblyopia.

The control group was recruited through the Academy for Lifelong Learning program at Carnegie Mellon University and consisted of 10 subjects (3 males, 7 females), with a mean age of 71 years and a mean educational level of 15.6 years. Three of the control subjects were tested with glasses. No subject had any history of neurological disease and none showed evidence of hemispatial neglect on a bedside battery of neglect [14].

The patient group consisted of 5 right-handed males, ranging in age from 22–67 years. All wore glasses but only one required glasses for this testing situation. The

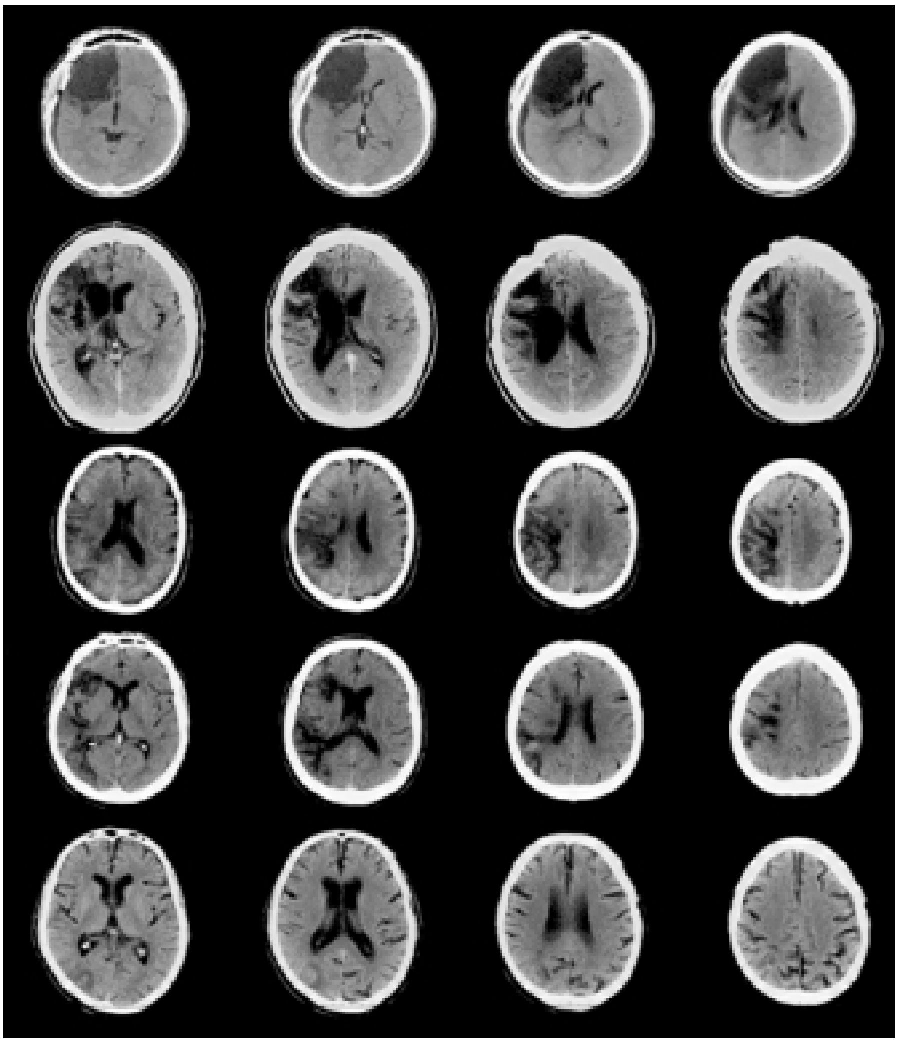

Fig. 1. MRI scans for the 5 patients, in order from top to bottom, depicting the location and extent of each subject's lesion. Left of the scan is the right side of brain, using radiological convention.

lesion sites of the patients is shown in Fig. 1 and the autobiographical data as well as neglect and lesion details are included in Table 1. Patients 3, 4 and 5 have lesions directly implicating parietal cortex, as is evident from their scans. Patient 1 has some parietal damage although it is not extensive and Patient 2 has extensive thalamic damage, thereby de-afferenting parietal cortex and essentially precluding it from contributing to behavior. No patient was hemianopic. The presence of neglect was defined on a bedside battery (Sunnybrook bedside battery) consisting of standardized tests of line bisection, target cancellation, drawing and copying [14]. Performance of the patients on this battery is scored in contrast with a large group of normal control subjects. On this battery, the lower the score, the more normal is performance, and the maximum score of 146 indicates profound neglect. As is evident from this, all five patients have fairly significant neglect, ranging upwards from 97 points. These patients have participated in previous studies and the reader is referred to [10,66] for further details about their performance. Note that our concern here is not in differentiating between patients with frontal and parietal lesions but rather in exploring the metrics and dynamics of eye movements of patients with posterior lesions, affecting parietal function.

### 5.2. Apparatus

The experimental apparatus was located in a windowless room, with the walls and ceiling painted black.

Table 1
Autobiographical and lesion data for the five brain-damaged patients

| Patients | Age | Lesion site | Volume | % 39[a] | % 40[a] | Time test[b] | Neg score[c] |
|---|---|---|---|---|---|---|---|
| 1 RD | 22 | Frontoparietal | 252 | None | <10 | 7 | 107 |
| 2 JM | 52 | Thalamus | 126 | None | None | 48 | 111 |
| 3 JB | 66 | Parietal | 123 | 50–89 | >90 | 17 | 103 |
| 4 JS | 67 | Parietal | 166 | 50–89 | 50–89 | 4 | 97 |
| 5 RB | 63 | Parietal | 114 | 10–49 | <10 | 33 | 114 |

[a] Percentage of Brodmann parietal areas 39 and 40 involved in the lesion. Only JB and JS had a lesion involving parietal areas 5 and 7 as well.
[b] Time of testing, post onset in months.
[c] Neglect score (maximum = 146).

There were no lights in the room except two dim night-lights, one on each side of the subject, located close to floor level. The apparatus consisted of a table, a head/chinrest mounted on one side of the table, and a chair on castors, all facing an array of light emitting diodes (LEDs), located along an arc of radius 1m, and centered at the eye level of the chinrest. The electronic apparatus consisted of two parts. The first system consisted of the LED array and of the electronic equipment required to control their activation: the LEDs were controlled from an IBM-PC by reading from a file the sequence and duration of activation of the LEDs in each experimental condition. The second system consisted of the eye movement recording apparatus, which was connected to electro-oculography (EOG) electrodes placed around the subject's eyes. This apparatus included an IBM-PC equipped with dedicated software for the acquisition of EOG data. The sampling rate of the system was 500 Hz per channel. The analysis of the data was performed off-line on an IBM-PC, using a Microsoft Windows-based software program. This software allows for trial-based calibration of the eye-movement recordings (i.e., the conversion from voltage data to eye position in terms of degrees of visual angle) and for the automatic computation of a host of dependent measures including the saccadic reaction time, amplitude, terminal accuracy, velocity, duration, peak acceleration, and peak deceleration of the saccade. Trial-by-trial calibration was chosen to reduce the effects of signal drift or head/trunk movements which might have occurred during the recording of the eye movements. This is especially important as, in some cases, neglect patients exhibit a tendency to drift rightwards during a trial. The accuracy of the system has been tested in the past and saccades of 1 degree in amplitude are easily identifiable as well as the large majority of saccades of 0.75 deg. In terms of the accuracy of estimates of the accuracy of eye fixation, accuracy was within 0.25 degrees of visual angle.

### *5.3. Procedure*

The subjects were first given an eye examination (using a stereo optical industrial vision tester) to document their eyesight and to determine, for those wearing glasses, whether testing had to be performed with or without glasses. Subjects with Snellen Equivalents of 20/100 or better were tested without glasses.

After the eye exam, the skin around the subject's eyes was cleaned and the EOG electrodes were applied: 2 silver chloride electrodes with adhesive tape rings were placed to the left and right of each eye (to monitor eye movements), 2 electrodes were placed above and below the left eye (to monitor blinks), and 1 electrode was positioned on the center of the forehead (ground), for a total of 7 electrodes. The electrode impedances were tested to ensure a proper electrical connection.

Subjects were seated in a chair on castors which were rolled under the table until the chest of the subject came in contact with the edge of the table. Subjects' chins were secured with a strap in a head/chinrest to minimize head movements and a strap going around the table secured the chair to the table to prevent any movements of the chair. Once the subject was properly oriented, calibration of the equipment was performed. A random sequence of activation of the LEDs corresponding to the selected target locations was initiated and the subject moved the eyes to each new location as soon as an LED was activated: this was done to ensure that there was no fundamental oculomotor deficit, that the subject could move his eyes to all locations and that the equipment was correctly recording the corresponding eye movements. This was particularly important in the case of the neglect patients to accustom them to the presence of a target on the contralesional side and to verify that they could move their eyes to these left-sided targets. In those cases where calibration was not easily achieved initially, we verbally instructed patients to be sure to look for contralesional targets.

On each experimental trial, the subject was instructed to keep his gaze on the fixation LED until the LED at one of the target locations was activated; at this point, the subject had to move his eyes to the target LED and keep his gaze there until both the fixation and target LEDs were turned off. This method is equivalent to the 'overlap' paradigm which has been shown to be highly sensitive to eliciting hemispatial neglect [47,82, 83]. Each trial had the following temporal sequence: at the beginning of a new trial, the fixation light appeared, flashed intermittently and was accompanied by an acoustic cue emitted from a speaker placed behind the LED arc and out of sight; after an 800 ms interval, the fixation light became steady and the acoustic cue stopped; after a variable (200, 800 or 1400 ms) time interval (SOA), the target light appeared; after a 1200 ms time interval (overlap), both lights (fixation and target) were turned off; finally, after a 2000 ms time interval (intertrial interval), the fixation light appeared again to start another trial. Subjects were given an opportunity to rest between blocks for as long as they needed.

There were three conditions, each involving a different starting position of the eyes. In all conditions, the subjects' head and trunk were aligned with 0 degrees defined with respect to the LED arc. The three conditions were:

a. *eyes center* (*EC*)*:* the subject fixated 0 degrees of the LED arc and the targets were sampled at 5, 10 and 15 degrees to the left and right of this 0 degree midline;
b. *eyes right* (*ER*)*:* fixation was fixed at 15 degrees to the right at the start of each trial and targets at 5 and 10 degrees on the right of space but to the left of the line of fixation were sampled; and
c. *eyes left* (*EL*)*:* fixation was fixed at 15 degrees to the left at the start of each trial and targets at 5 and 10 degrees on the left side of space but to the right of the line of fixation were sampled.

The combination of these three arrangements provides an orthogonal crossing of the two factors of target side (left, right) with saccadic direction (left, right). Trials were blocked according to start position of the eye but angle was probed randomly within a block with 9 trials per angle. In all cases, the EC condition was run first and then the order of the other two conditions was counterbalanced. Six control subjects completed 2 blocks of each condition while four completed one block of each condition. As much data was collected from each patient as possible: patients 1, 3, 4 and 5 completed 3 replications of each condition and patient 2 completed 5 replications. Note that we also sampled the 15 degree targets in the EC condition.

The raw eye-movement data were collected on-line on a Bernouilli disk and transferred off-line for analysis. The analysis software allowed for calibration of the individual saccades and for the automatic computation of the various dependent measures. Each trial was manually edited to review the results of the automated analysis. Although the eye movements of both eyes were recorded, dependent measures were only derived from the tracing from one eye. Both eye recordings were examined and then the one with lower noise levels or the one indicated as the better eye by the subject was chosen for this purpose (although no subject was amblyopic).

In general, trials were considered technically valid or acceptable when fixation was maintained for at least 100 ms prior to the onset of the target light and when the saccade occurred at least 70 ms after target onset. Trials where fixation was not maintained, when the saccade occurred prior to 70 ms (anticipatory saccade), when there were too many blinks, or when calibration was not possible were removed from the data. We then classified the remaining trials into those on which there was more than a single saccade (multi-step trials), those on which there was a single saccade but it was inaccurate in terminal position, those on which there was a direction error, and those on which there was no response. The remaining trials were accurate, single step trials and we used these to analyse the temporal properties of the saccade. The multi-step, single step, inaccurate saccades and directional errors might arise from a problem in correctly executing the saccades and any directional asymmetry (left, right) in these trials might be taken as evidence for an execution deficit.

The temporal measures are intended to provide details of both the planning and motor aspects of the eye movements and include saccadic reaction time (SRT, defined as the time between the onset of the target and the time the eyes start moving where velocity exceeds $10°$/s), duration of the saccade (defined as the time between the start of the eye movement and the time the eyes reach their terminal position, terminal position (where velocity falls below $10°$/s), and peak velocity.[1]

[1]The velocity vector is computed as follows: for $(i = 1;\ i < n-1; i{+}{+})$ $\mathrm{vel}[i] = (\mathrm{pos}[i+1] - \mathrm{pos}[i-1])/2.0$ where pos[] is the eye position vector. The values in the position vector represent the output of the A/D converter (= digitized volts readings). To compute the peak velocity, first the maximum velocity is computed ($\max v$) and then the peak velocity in degrees per second is computed as: peak$v$ = $\max v *$ sampling_rate / calibr_coeff where the sampling rate = 500 Hz and the calibr_coeff is volts/degree.

The SRT or initiation time is assumed to reflect the planning component whereas the others reflect the transport or movement (execution) of the saccade. For each of these dependent measures, analyses of variance are conducted with side (targets on left or right) and eccentricity (5,10 degrees) as within-subject factors. Where significant interactions are present, post hoc analyses are conducted using Tukey tests with $\alpha = 0.05$. Only significant interactions are reported. The data reported here form a subset of a larger data set collected in order to examine the influence of different frames of reference on saccadic performance in patients with neglect (see [10] for analyses of SRT to the left and right targets when frames of reference of the eyes, head, and trunk are orthogonally manipulated).

### 5.4. *Results*

To determine whether the patients are impaired at both initiating and executing contralateral saccades, relative to the control subjects, we first present the data from the EC condition alone. Thereafter, we consider the additional contribution of orbital position to the saccadic behavior of the patients and analyse the data from the manipulation of eye position.

### 5.5. *Eye-centered condition*

In the EC condition, we use data from targets appearing at all angles (5, 10 and 15) to examine the eye movement deficit. Invalid trials, as defined above, constituted 21% of the data from the patients and 3.4% of the data from the control subjects. For the remaining trials, we first report the results for all those trial types on which the temporal parameters cannot be obtained. This includes, for the patients, the results of the multi-step (6%), inaccurately localized (19.7%), direction error (7%), and omission (11%) trials. Afterwards, we analyse the latencies and velocities of the remaining 36% of the trials on which a single, well localized saccade was made.

*a. Multi-step trials:* This refers to those trials on which more than one saccade was made. In general, there is an initial hypometric or hypermetric saccade followed by additional corrective saccade/s. Table 2 includes the number of multi-step trials for each of the five patients, as well as the mean across the 10 control subjects. An ANOVA with group as a between-subject factor and side (left, right) and target eccentricity $(5, 10, 15)$ as within- subject factors reveals a significant main effect of group, $(F(1,13) = 13.3,\ p < 0.01)$, with the patients making more multi-step saccade trials than the controls. There was also a main effect of target eccentricity, $(F(2,2) = 10.6,\ p < 0.001)$, with more multi-step saccades as target eccentricity increased, but this is especially true for the patients $(F(2,26) = 7.1,\ p < 0.01)$. Most importantly, there is no two-way interaction of group x side, $(F(1,13) = 0.09,\ p > 0.5)$ indicating that the patients do not show a difference in the number of such trials on the left or right. There is also no three-way interaction between group, side and target eccentricity, $(F(2,26) = 0.1,\ p > 0.5)$, further indicating equivalent patterns of performance for both groups as a function of side and eccentricity.

*b. Inaccurate trials* were those that consisted of a single saccade only and were technically valid but did not fall close to the target position, being either hypermetric or hypometric but with no additional corrective saccade following the inaccurate initial saccade. Because saccadic precision is diminished in older (non-neurological) individuals compared with younger individuals [2] and in cases where the target does not need to be discriminated, we needed to establish an accuracy criterion in the normal subjects against which to compare the patients. We did so by defining a boundary around the target location in which 95% of the control data fell:

mean(saccade end $-$ final angle) $+/-$ 2
*sqrt(var(saccade end - final angle))

and this yielded an interval around the final angle.[2] Thus, trials which ended more than $+$3.018515 degree or less than $-$3.324983 degrees from the target were considered inaccurate. Of the 5% of the control data that did not fall in this interval, 60% were hypometric and the remaining 40% were hypermetric. For the patients, 80.3% of the trials fell within this specified bound and, of the remaining outlying trials, 87% were hypometric. Thus, for the inaccurate errors, there was obviously a main effect of group as the boundary was defined with respect to normal control performance, $(F(1,13) = 0.182,\ p < 0.05)$. There was also a

[2] We verified that the results did not differ if we chose a different boundary for deciding on the tolerance around the terminal point of the saccade. In this alternative procedure, we disregarded the performance of the normal subjects and, instead, simply set a fixed boundary of 2.5 degrees around each target position. Thus, for a target located at $+5$ degrees, only those saccades that fell between $+2.5$ and $+7.5$ were considered acceptable. The results did not differ from those obtained with the first method, confirming the robustness of the exclusion procedure.

Table 2
Number of multi-step trials for patients and controls (mean) as a function of side and distance

| Patients | Left 15° | Left 10° | Left 5° | Right 5° | Right 10° | Right 15° |
|---|---|---|---|---|---|---|
| | 1 | 6 | 10 | 4 | 8 | 9 |
| | 10 | 12 | 15 | 6 | 25 | 33 |
| | 0 | 0 | 2 | 5 | 7 | 14 |
| | 12 | 18 | 21 | 2 | 1 | 1 |
| | 0 | 2 | 2 | 13 | 10 | 9 |
| Controls | 1.6 | 1.7 | 2.1 | 2.8 | 3.3 | 3.8 |

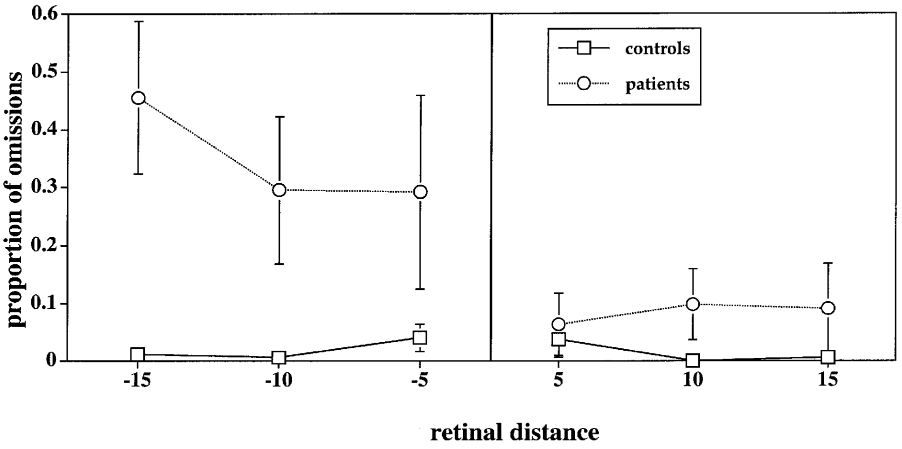

Fig. 2. Proportion of omission errors for patients and control subjects as a function of side and target distance.

significant effect of target eccentricity, $(F(2,26) = 3.2,\ p = 0.05)$, and an interaction between group x target eccentricity, $(F(2,26) = 3.36,\ p = 0.05)$, with the patients making more inaccurate saccades as eccentricity increased, collapsed across fields, relative to the control subjects. Note that, as with the multi-step saccades, there are no interactions with side (all $F < 1$), indicating that there is no specifically contralesional impairment in these inaccurate trials for the patients over and above the control subjects.

*c. Direction errors* refer to those trials in which a saccade was properly executed but on which the saccade was made in the direction opposite the target location. There were no statistically significant differences between the two groups for the direction errors, nor between side nor target distance and no interactions at all (all $F < 1$).

*d. Omissions* consisted of trials on which there was no eye movement (the eyes "locked" on fixation), trials where fixation was maintained for some time but then the eyes started drifting off fixation or oscillating around it and no true saccade was made, and trials where the saccade occurred more than 1 second after target onset. Overall, there were few omissions which is not surprising given that the subjects knew that there was a target on every trial and it was simply a matter of time until the target was found. Despite the small number of trials, the ANOVAs yielded a significant main effect for group $(F(1,13) = 9.7,\ p < 0.001)$ with the patients making more omissions overall than the control subjects (see Fig. 2). There was a significant main effect of target side, $(F(1,13) = 13.5,\ p < 0.001)$, with more errors on the left than on the right, collapsed across groups. There was also a significant interaction of group x target eccentricity $(F(2,26) = 3.3,\ p = 0.05)$, with the patients showing a greater increase in error rate as eccentricity increases, collapsed across both target sides, compared with the control subjects. Finally, and perhaps most relevant, there was a significant interaction of group x target side, $(F(1,13) = 24.0,\ p < 0.0001)$, with more left-sided errors for the patients than controls and no difference between the two groups for targets on the right.

As is evident, the patients' left-sided errors do not differ statistically as a function of distance or eccentricity (although there is a hint of this numerically), as one might have expected from studies which propose that a gradient of attention is observed in neglect patients [18,48,61,68,78]. The absence of a statistically significant gradient here might simply be a function of

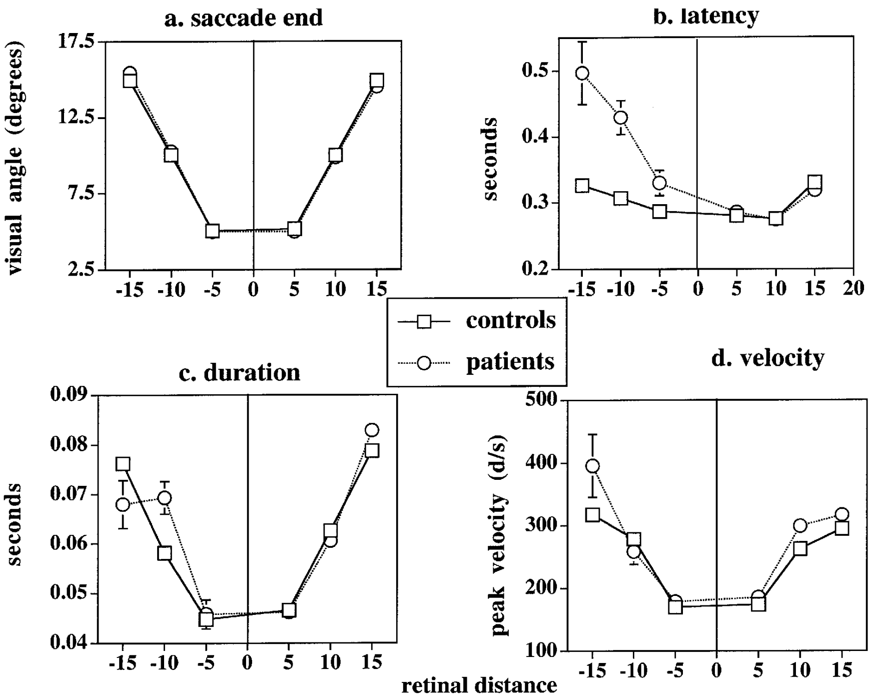

Fig. 3. Mean (and 1 SE) (a) saccade end, (b) latency, (c) duration and (d) velocity for eye movements for patients and controls subjects as a function of side and angle distance. Data from the eye-centered condition alone are presented here.

the nature of the task (target present on every trial) and the low error rate. The small number of errors reduces the statistical power to detect the small differences between target angles. The reaction time data are more informative on this point, as shown below.

*e. Single step, accurate trials.* The ANOVAs performed on the other dependent measures include only the single step, accurate trials. Although the number of data points for the patients is now reduced to a total of 36% of the data, these are 'pure' trials and would be the most likely to reveal any deviations from the normal pattern. The results of these temporal analyses are shown in Figs 3(a)—(d). Note that on Fig. 3, standard error bars are included but are not always visible because of the compression of the y-axis scale.

We first verified that, for the correct trials, the terminal position of the saccades was equivalent across the two groups. This is critical as other measures such as duration and velocity are strongly dependent on distance. Although we had previously removed trials from the patients which fell beyond a specified boundary around the target (classified as inaccurate trials, see above), we wanted to ensure that within this bound, the end points of the saccades were not different for the two groups. Using saccade end point (defined in visual angle) as the dependent measure, target distance and side as within-subject factors and group as a between-subject factor, we see a significant two-way interaction of target eccentricity x target side ($F(2,13) = 5.6$, $p < 0.01$), as well as main effects of target side ($F(1,13) = 9.7$, $p < 0.005$) and eccentricity ($F(1,13) = 7.1$, $p < 0.0001$). These findings, shown in Fig. 3(a), reflect greater saccadic precision for smaller than larger angles and slightly less precision on the left than right, especially with the larger angles, all collapsed across the two groups. Importantly, there is no main effect of group nor an interaction of group with any other factor (all $F < 1$). That the two groups have equivalent end point accuracy for the correct trials is critical for the remaining analyses.

Having established the group equivalence for saccadic termination, we then conducted an ANOVA with saccadic reaction time (SRT) as the dependent measure. We used a logarithmic transformation of the data to adjust for unequal error variances and non-normality of the distributions. As above, group served as the between-subject factor and target eccentricity $(5, 10, 15)$ and side (left, right) served as within-subject factors. Although the ANOVAs are performed on the log latency, we present the cell means on the original scale for ease of interpretation and comparison with the existing literature. The standard errors from the log analysis are transformed back to the original scale using the delta method. The results from this analysis are shown in Fig. 3(b).

As might be expected, all main effects were significant. Patients were 55 ms slower than the control subjects, collapsed across all targets, $(F(1,13) = 26.3,\ p < 0.0001)$. Collapsed across groups, latency increased with target eccentricity $(F(2,13) = 19.7,\ p < 0.0001)$. Latencies were also significantly slower for left than right targets $(F(1,13) = 48.7,\ p < 0.0001)$ with a 66 ms difference between them. There was also a significant two-way interaction between target distance x side; although latencies were slower with more distant targets, collapsed across group, this was disproportionately so for the left field, $(F(2,13) = 6.7,\ p < 0.01)$. Of more importance, however, is the two-way interaction between group $x$ target side with the patients performing more slowly than the controls on the left but not on the right, $(F(1,13) = 30.9, p < 0.0001)$. Most important, however, is the significant three-way interaction between group, target eccentricity and side; this arises because the latencies for the patients are disproportionately slow as target eccentricity increases, relative to the control subjects, and this is only so for targets on the left.[3] Specifically, the mean latency for $-10°$ and $-15°$ for the patients are not significantly different from each other (although there is a numerical trend in this direction) and these differ significantly from latencies at all other target angles. For the control subjects, latencies for 15° and $-15°$ were slower than for the smaller angles on both the left and right and latencies at 15° were significantly slower than for $-15°$.

The same ANOVA was conducted with duration of the saccade using the reciprocal of the duration (1/time) as a stable dependent measure (see Fig. 3(c), with data transformed back to original scale). The analysis revealed no main effect of group, $(F(1,13) = 0.37,\ p > 0.1)$. Across groups, durations of eye movements were, however, marginally longer in the right than left field $(F(1,13) = 3.16,\ p = 0.07)$ and longer as target eccentricity increased $(F(2,13) = 163.1,\ p < 0.0001)$. There was also a two-way interaction between field and target eccentricity revealing significantly longer duration times for the target at 15° than for any other angle. These findings are all qualified by the presence of a three-way interaction between group x target distance x target side $(F(2,13) = 6.9,\ p < 0.001)$. The pairwise comparisons, however, yield a rather strange and unexpected pattern: at $-15°$, the control subjects take significantly longer to execute a saccade than do the patients whereas at $-10°$ and at 15°, the patients take significantly longer than the control subjects. A few other aspects of the patients' data are worth noting at this stage: the patients take longer to move their eyes to 15° than they do to a target at $-15°$, they show no difference in duration between $-15°$ and $-10°$ and durations are not significantly different between $-5°$ and 5°. These findings speak against a specific problem in the patients' ability to execute leftward versus rightward saccades.

The identical and final ANOVA was conducted using log peak velocity as the dependent measure and the results (again, in the original scale with transformed standard error bars) are shown in Fig. 3(d). A main effect of target eccentricity was obtained, $(F(2,13) = 130.3,\ p < 0.0001)$ with faster velocity for longer distances, as expected, although this held more strongly for targets on the left than the right, $(F(1,2) = 2.9,\ p = 0.05)$, and marginally more so for the patients than for the control subjects, $(F(1,2) = 2.72,\ p = 0.06)$. There was also a significant interaction between target side x group, $(F1,13) = 4.38,\ p < 0.05)$, with a consistently greater difference between the patients and controls for targets on the right than on the left. Importantly, however, there is no significant three-way interaction, $(F(2,13) = 1.11, p > 0.3)$; thus, to the extent that the groups differ in their velocities to the left and right, as shown in the marginal two-way interaction, this does not differ across target eccentricity and the pattern does not conform to the pattern one would expect to be associated with neglect (as is the case in the latency data).

### 5.6. *Influence of orbital position of the eye on saccadic target acquisition*

The next set of analyses are concerned with the interaction of target side and eccentricity with saccadic direction (orbital position). These analyses include the data from the EC, as analysed above, as well as from the ER and EL conditions for targets at 5 and 10 degrees (to the left and right of fixation, respectively) for the full factorial comparison of target eccentricity and

[3]Note that the latency values for the EC condition here differ slightly from those reported in Behrmann et al. (2002) for the baseline condition even though they are the same data. The difference arises because here we have trimmed the data and have only used accurate trials because of our concern for the duration and velocity analyses. These same considerations were not critical in the other paper. The fundamental results are not affected by this difference.

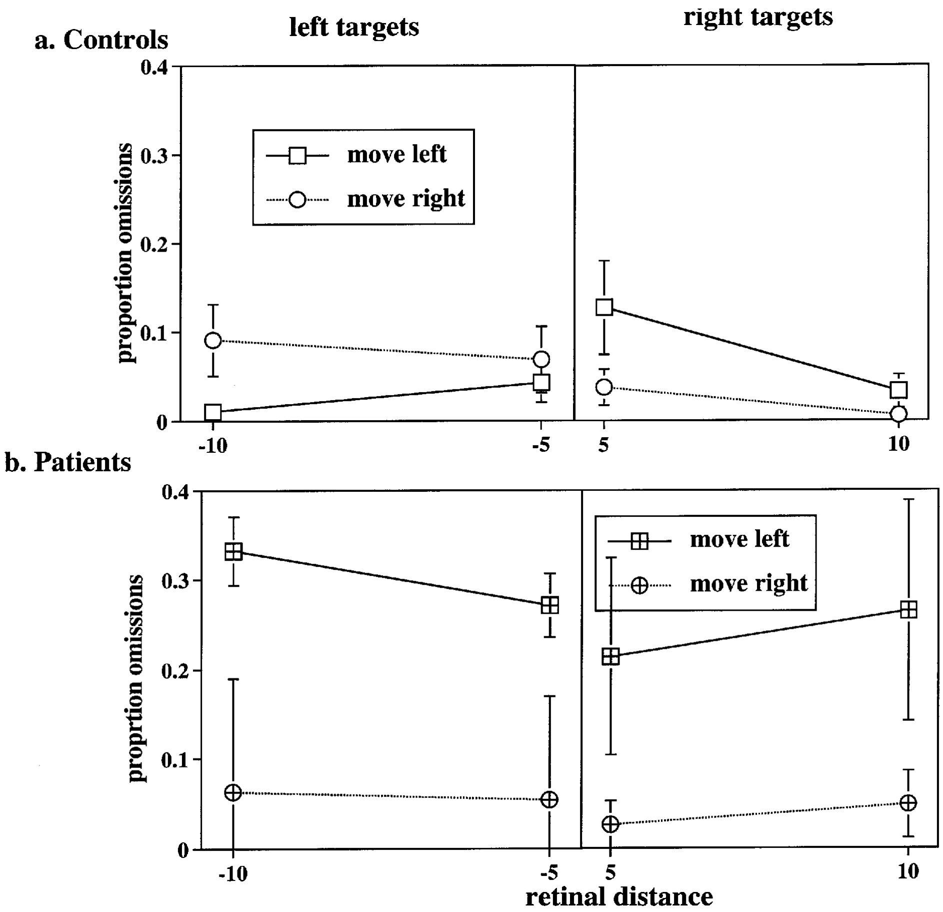

Fig. 4. Proportion of omission errors (and 1 SE) as a function of target distance for (a) controls and (b) patients for leftward and rightward saccades to targets on the left and right. These data include the data from the eye-centered, eye right and eye left conditions.

eye position. For the patients, the single step, accurate trials constituted 31.6% of the data set. The omission trials constituted 16.2% of the data, whereas the multi-step, inaccurate and direction errors constituted 5, 4, and 3%, respectively. Trials which were technically invalid constituted 40% of the patient data set. The increased percentage of technically invalid trials, relative to the EC condition alone, might reflect the patient's fatigue as the EL and ER conditions are always run after the EC condition, and patients may have been less able to hold fixation by this stage of the experiment. Even though for some patients the data were collected in multiple sessions, the EC condition was always run before the others. Note, however, that there are more accurate trials in this analysis relative to the EC condition alone and, thus, roughly the same number of trials is available for kinematic analysis, as above. Because the omission trials revealed the critical side x group interaction in the previous analysis, we examine only those trials in addition to the single step, accurate trials on which the kinematic measures are obtained.

*a. Omission trials:* the results of the analysis of the omission trials are shown in Fig. 4 with the control subject data appearing above the patient data and separate panels for left and right targets, as a function of saccade direction. This same format of graphic display will be used for all subsequent analyses. An ANOVA with target side (left/right), direction of saccades (move left, move right) and target eccentricity (5,10) as within-subject factors and group as a between-subjects factor revealed a significant effect of saccadic direction with both groups making more errors when making eye movements leftwards than rightwards ($F(1,2) = 16.8$, $p < 0.0001$). However, this was qualified by an interaction with group ($F(1,2) = 13.5$, $p < 0.001$), reflecting the greater overall errors for patients in leftwards than rightwards saccades, relative to the control subjects. There was a significant main effect of group

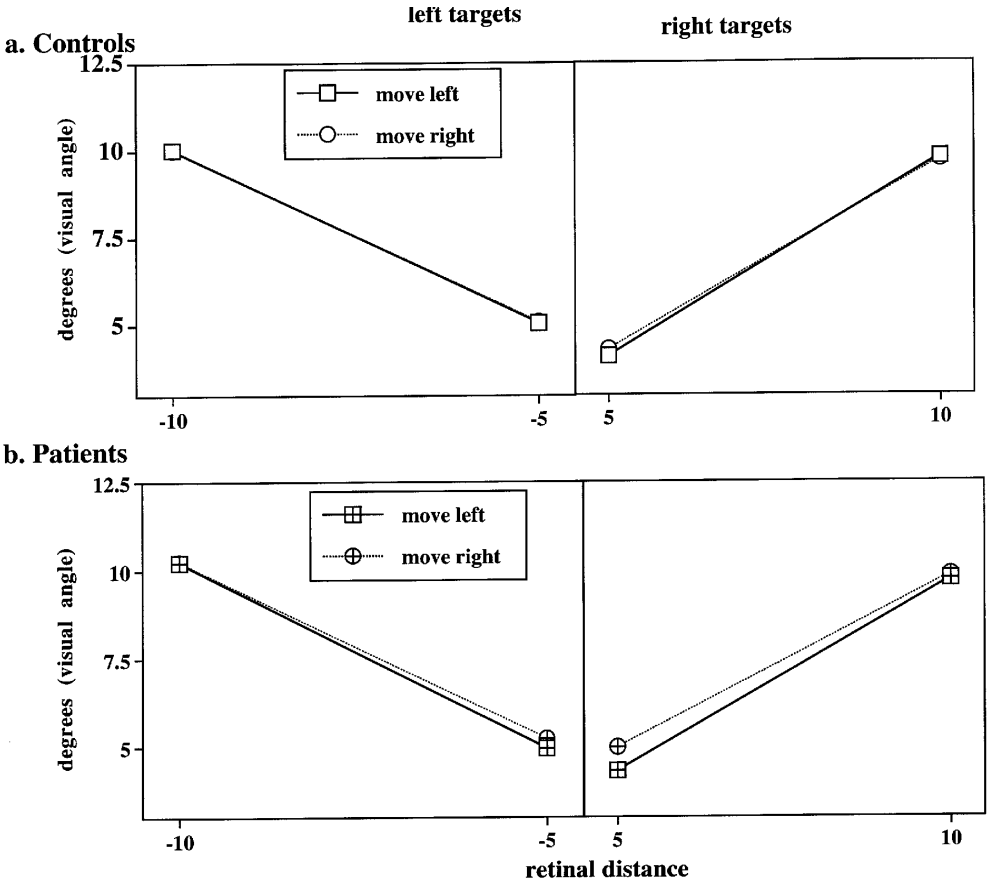

Fig. 5. Saccade end (and 1 SE), plotted in visual angle, as a function of target distance for (a) controls and (b) patients for leftward and rightward saccades to targets on the left and right. These data include the data from the eye-centered, eye right and eye left conditions.

with the patients making more errors than their normal counterparts ($F(1,2) = 4.16$, $p < 0.04$). There was also a marginally significant effect of target eccentricity with a trend towards more errors as eccentricity increased ($F(1,2) = 3.15$, $p = 0.07$), and this was disproportionately true for the patients relative to the controls, ($F(1,2) = 9.05$, $p < 0.005$), and more so for leftwards than rightward saccades ($F(1,2) = 8.92, p < 0.005$). Most important is that there is a three-way interaction of target side, direction of saccade and group ($F(1,2) = 10.4$, $p < 0.002$). This analysis reveals that for the patients, when the saccades are made leftwards, there are more errors for left than right targets; when saccades are made rightwards, however, there is no difference between left and right targets. This pattern holds true across target distance. There are no statistically significant differential patterns for left compared with right targets as a function of orbital direction in the normal controls.

*b. Single step, accurate trials* were analysed in an ANOVA with group as a between-subject variable and target eccentricity (defined retinally; 5, 10 degrees), target side (left, right) and saccade direction (move left, move right) as within-subjects factors. We first ensured that the terminal point of the saccade was equivalent across the two groups before examining the other dependent measures. As before, we used the boundary defined by normal subjects to classify trials as accurate or inaccurate. The following analysis is only on those trials which fall within the boundary specified by the control data. Using saccade end (in visual angle) as the dependent measure, we see significant main effects of distance ($F(1,2) = 11683$, $p < 0.0001$), target ($F(1,2) = 10.2$, $p < 0.002$), saccade direction ($F(1,2) = 21.9$, $p < 0.0001$) and of group ($F(1,2) = 5.1$, $p < 0.03$). Some of these effects are subsumed in higher-order interactions, specifically an interaction of group x target side x saccade direction ($F(1,2) = 5.44$, $p < 0.0198$). These findings are

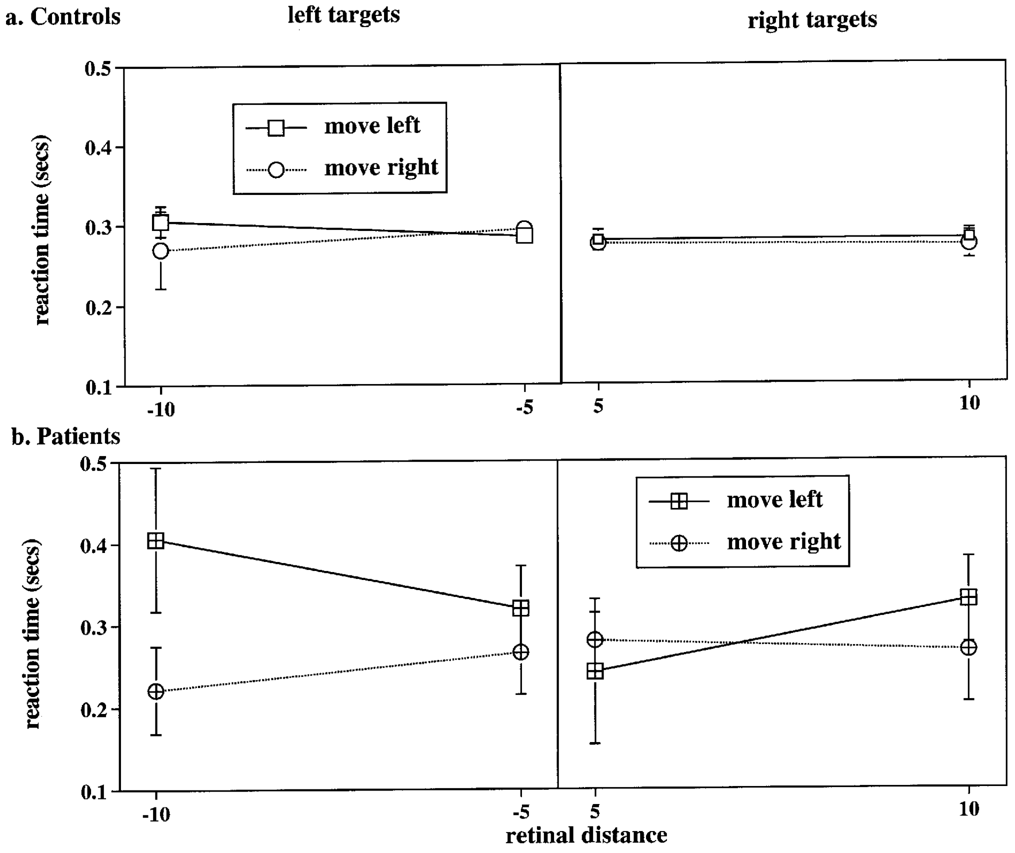

Fig. 6. Saccadic reaction time (and 1 SE) in seconds as a function of target distance for the (a) control subjects and (b) patients for leftward and rightward saccades for targets to the left and right. These data include the data from the eye-centered, eye right and eye left conditions.

shown in Fig. 5. As is evident, for the most part, both groups of subjects are precise in making the saccade to the correct destination. The significant interaction arises because patients are slightly less accurate (overshooting) in making saccades to targets on the right when moving rightwards (from EC position) than in any other conditions and this holds equally across target eccentricity. The effect is small, however, and is not an obvious consequence of the left-sided neglect. The control subjects show no differences as a function of any of the variables. This analysis is re-assuring and suggests that we can go on to evaluate the temporal characteristics of the saccades without concern for a difference between the groups in the extent of the saccade.

We then examined the latency data and this analysis is critical as we see a deficit in initiating saccades to positions on the left compared with the right in the EC condition reported above. We performed a logarithmic transformation on the initiation times to adjust for unequal error variances and the non-normality of the distributions. An ANOVA with the same design as that used to analyse the above data was conducted with log latency as the dependent measure. The results are shown in Fig. 6. This analysis revealed a significant effect of target side ($F(1, 13) = 4.05$, $p < 0.05$) and saccade direction ($F(1, 13) = 23.98$, $p < 0.0001$) but no main effects for group nor for target eccentricity (both $F < 1$). There were also significant two-way interactions between target eccentricity x saccade direction, ($F1, 13) = 24.06$, $p < 0.0001$), group x saccade direction, ($F(1, 13) = 12.3$, $p < 0.001$) and a marginal effect of group x target eccentricity, ($F(1, 13) = 3.1$, $p = 0.07$). The three-way interaction of group x target side x saccade direction is also significant, ($F(1, 13) = 12.02$, $p < 0.001$) but all of these higher-order interactions are subsumed by the four-way interaction of target eccentricity (defined retinally; 5, 10 degrees), target side (left, right) and saccade direction (move left, move right) with group (patients, controls), ($F(2, 13) = 5.01$, $p < 0.01$).

To facilitate comparisons between the groups, we have plotted the data for left and right targets separately for the two groups as a function of target distance. We have plotted the data in the original scale for ease of understanding and comparison with other data and we have conducted post hoc Tukey tests with $p < 0.05$. As is evident from a comparison of these plots (see Fig. 6), there were no differences in the control subjects in the time to initiate a saccade to the left or right at either distance and from either saccadic direction. This was not the case for the patients. For right targets, there are no differences to initiate saccades leftwards or rightwards, no differential effect of target distance and no difference from the control subjects. The most interesting finding is for left targets; leftward movements take much longer than rightward movements, especially for more distant targets and, this is also true when compared to the control data. In fact, the latency to initiate leftward saccades to targets located at 10 degrees on the left is the slowest condition compared to all other cells. There is also a small but significant slowing for patients over controls for targets at 5 degrees on the left when the movement is leftward.

To examine whether this slowing in initiating leftward saccades to leftward targets also extends to a problem in executing the saccades, we conducted the same ANOVA with duration as the dependent measure of interest. As before, we used the reciprocal of the duration as a stable measure of time and the results of this analysis are shown in Fig. 7. There was a significant main effect of retinal eccentricity, $(F(1,13) = 210.3,\ p < 0.0001)$ which is not surprising given that the farther the distance, the longer the duration of the saccade is likely to be. There was also a significant effect of target side $(F(1,13) = 4.32,\ p < 0.05)$, with longer durations for left than right targets. Leftward eye movements were also significantly slower than rightward movements, $(F(1,13) = 13.3,\ p < 0.0001)$ and there was a main effect of group, $(F(1,13) = 5.11,\ p > 0.05)$, with patients being overall slower than the control subjects. There was a significant effect of target eccentricity x side, $(F(1,13) = 5.65,\ p < 0.02)$, and this arises because saccades to targets at 10 degrees on the right are slowest of all trials. There were significant two-way interactions between group x saccadic direction, $(F(1,13) = 4.96,\ p < 0.05)$, with patients slower at making leftward than rightward saccades, collapsed across target side, relative to the controls, and between group x target side $(F(1,13) = 5.2,\ p < 0.05)$, with patients slower for left than right targets, relative to the controls. There was also a target eccentricity by saccadic direction, $(F(1,13) = 5.4,\ p < 0.05)$, with leftward saccades for larger eccentricities slower than the corresponding rightward saccades. There was also a significant three-way interaction of target eccentricity x target side x saccadic direction $(F(1,13) = 5.5,\ p = 0.01)$, reflecting the slower reaction times, across both groups for targets at 10 degrees on the right especially for leftward movements. Most relevant is that there are no higher-order interactions involving group [group x target side x saccadic direction $(F(1,13) = 3.1,\ p > 0.05)$; group x target side x saccadic direction x target eccentricity $(F(2,13) = 1.06,\ p > 0.3)$]. This means that, while the patients are slower at making leftward than rightward saccades, and are slower at making saccades to left than right targets, they are not significantly slowed in executing leftward saccades to left targets, relative to the controls, as is the case in the initiation (SRT) data.

Finally, we conducted the identical ANOVA on the velocity data (again, after transforming the values using a log scale), and the data are shown in Fig. 8 with degrees/sec on the y-axis. As expected, velocity increased for larger over smaller distances, $(F(1,13) = 357.4,\ p < 0.0001)$. Overall, velocities were faster for rightward than leftward movements, $(F(1,13) = 4.37,\ p < 0.05)$ and the other two main effects were not significant (target side and group, $F < 1$). There is also a marginally significant interaction of group x saccadic direction, $(F(1,13) = 3.6,\ p = 0.055)$, with patients showing faster velocities for rightward than leftward saccades but no difference between them in the control subjects. The only other significant interaction is between target eccentricity x target side x group, $(F(1,13) = 4.02,\ p < 0.05)$. This arises because velocities are a little faster for 10 degrees on the left than on the right for the patients whereas there is no difference as a function of side for the control group. There are no higher order interactions with saccadic direction and the four-way interaction of group x saccadic direction x target distance x target side is not significant, $(F(2,13) = 0.45,\ p > 0.5)$. The critical result here is that patients are not slower in their velocities to left targets when they make leftward saccades compared to the counterpart, right condition nor compared to the control subjects.

### 5.7. Discussion

Although it is well known that patients with hemispatial neglect exhibit a difference in their ability to make saccades to the contralesional versus ipsilesional side,

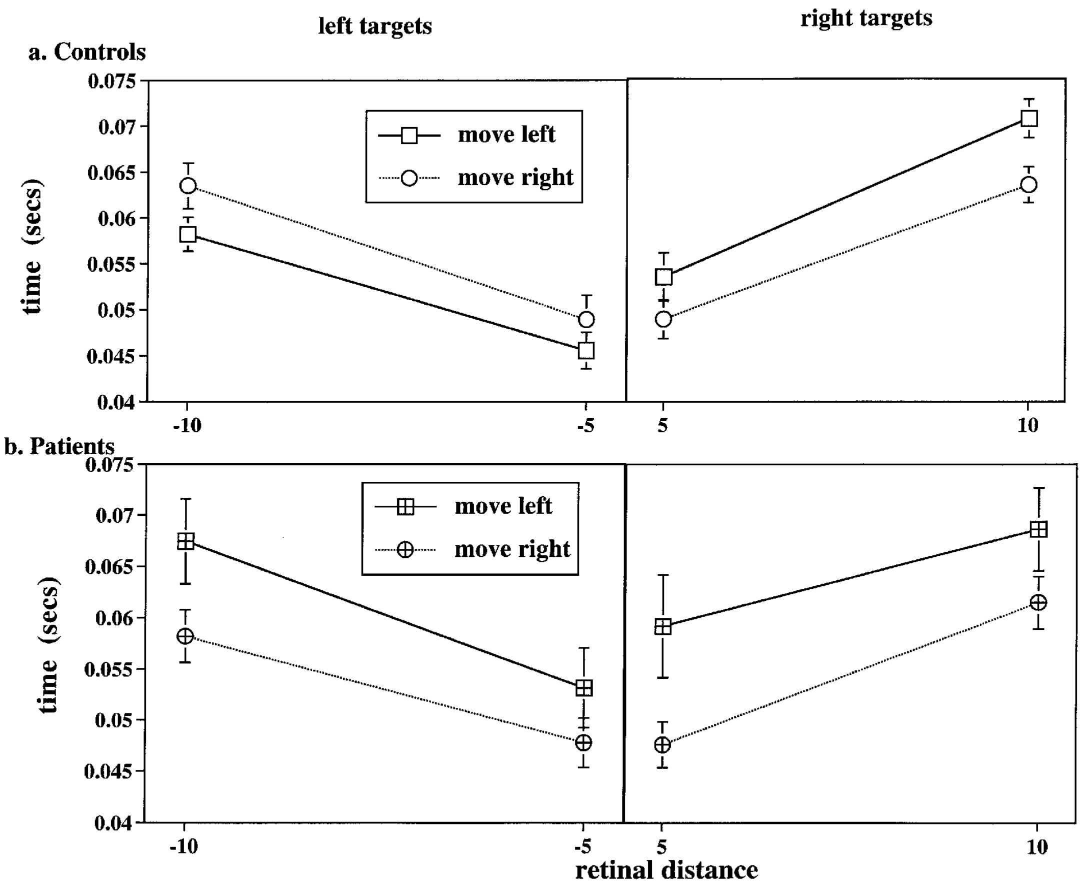

Fig. 7. Duration of saccade (and 1 SE) in seconds as a function of target distance for the (a) control subjects and (b) patients for leftward and rightward saccades for targets to the left and right. These data include the data from the eye-centered, eye right and eye left conditions.

the exact nature of this directional asymmetry is not well understood. To this end, we explored the performance of a group of neglect patients and their control counterparts in an oculomotor task in which we crossed two variables: side of target and direction of eye movement. The study had two major goals: to examine whether there is a problem in initiating and/or executing saccades to contralateral targets and, furthermore, whether this directional deficit is exacerbated when the saccade is directed leftwards compared to rightwards.

We first compared the eye movements of patients and control subjects when they made saccades from a starting point aligned with the midline of an LED array (also aligned with the midline of their head and trunk) to targets at three different eccentricities on the left and right sides. Note that because of this experimental set-up, the targets are also in the left and right visual fields. The results of this first analysis are fairly straightforward and suggest that the problem is one of planning and initiation rather than execution of the eye movement. We see a directional-specific problem for the patients, in that they failed to make an eye movement (omission trials) to targets on the left more often than on the right whereas the control subjects show no side difference. Furthermore, there is a trend such that the directional pattern in the patients is disproportionately exaggerated as target eccentricity increased. We also see a directional-specific pattern in the latency of the saccades on single step trials; patients are slower to initiate saccades to the left than to the right, with an increase in saccadic reaction time with increasing target eccentricity, whereas, again, there is no difference for the control subjects. This difficulty in initiating a saccade to contralateral targets is compatible with the many other existing results in the literature which document the slowed initiation of contralesional saccades [6,31,44].

The initiation asymmetry shown by the patients can be contrasted with their performance in executing the saccades. There is no difference between the patients

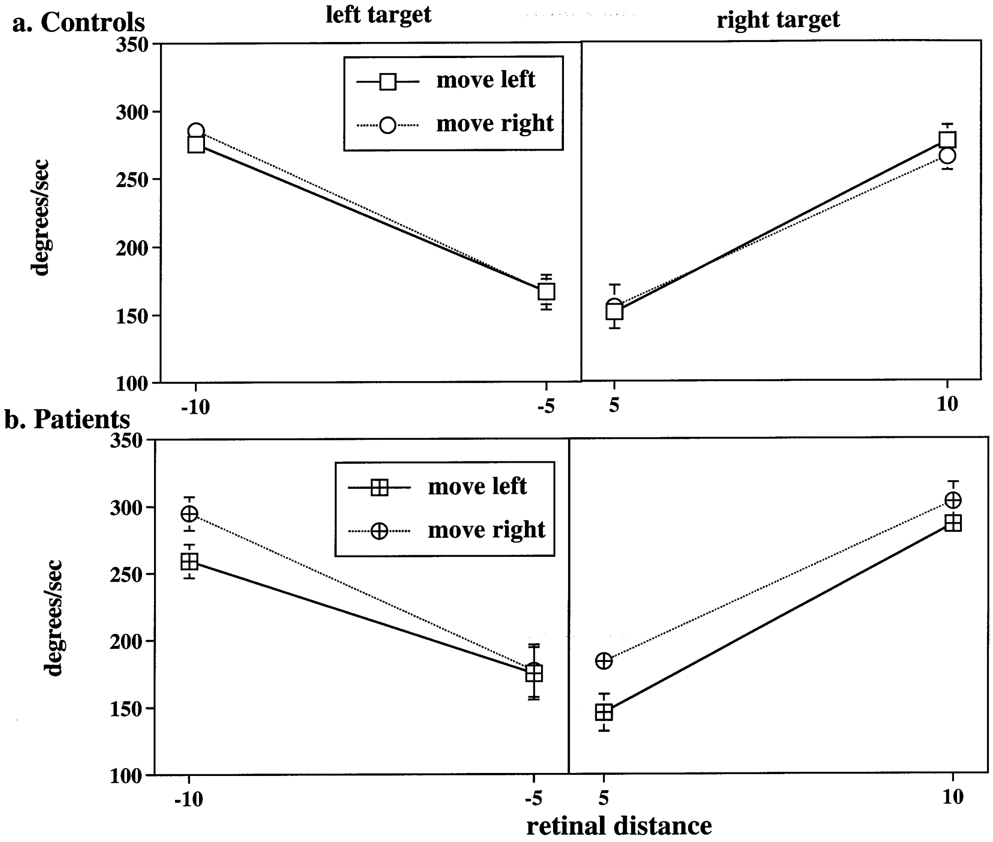

Fig. 8. Velocity (and 1 SE) in degrees/sec as a function of target distance for the (a) control subjects and (b) patients for leftward and rightward saccades for targets to the left and right. These data include the data from the eye-centered, eye right and eye left conditions.

and control subjects in their ability to move their eyes, as measured in the time taken to execute the saccade, or in the peak velocity of the saccade. Although there are some small differences between the two groups on these temporal measures, the patterns observed are quite different from what one might expect in hemispatial neglect. For example, the control subjects execute saccades more slowly to targets at 15 degrees on the left than do the patients and the patients make slower saccades to targets at 15 degrees on the right than on the left. The absence of clear differences between the patients and controls and the absence of a pattern that would correspond to what one might expect in neglect in the patients argues against an execution deficit in the oculomotor domain for the patients. Consistent with the absence of a contralesional saccade execution deficit, the patients also do not show more multi-step, single step, inaccurate (mostly hypometric) or directional error saccades than do the control subjects as a function of side although the patients do make more of each of these error saccades overall than do the control subjects.

The absence of a deficit in executing the saccade adds to the growing consensus that there is no direction-specific execution impairment in neglect patients with parietal lesions. Both Niemeier and Karnath [62] and Walker and Findlay [82] have also failed to observe a directional-specific execution impairment although, as in our case, the former report a reduction in amplitude of saccades in the patients in all directions, resulting in more inaccurate saccades than normals, whereas the latter find no obvious amplitude change at all. The results are also compatible with the observation by Duhamel and colleagues [28] that their patient with saccadic dysmetria following a large fronto-parietal lesion shows a normal correspondence between distance and velocity of saccades for both right and left targets.

Finally, these results are in line with the data from many, although not all, reaching studies, which indicate that neglect patients show directional hypokinesia (slowed initiation) but no directional bradykinesia

(slowed execution) in their manual action [45,49,75]. The results contrast, however, with other findings from the reaching literature which reveal problems in the spatial and temporal parameters of the reach itself in neglect patients. Some studies have shown that neglect patients show systematic deviations in their reach [32] as well as alterations in the acceleration/deceleration and velocity profiles [58]. We should note that, in our study, we have documented only the temporal characteristics of the eye movements rather than the spatial parameters of the saccade, aside from terminal destination of the saccade. Therefore, even though we see no evidence of an execution deficit, definitive evidence would also require quantification of the trajectory of the saccade itself.

Having demonstrated that the side of the target significantly influences the planning of the saccade, our second analysis incorporated the start position of the eye to examine whether target acquisition by the eyes is further impaired as a function of direction of saccade. With the addition of the data from the EL and ER conditions, we confirmed the patients' problem in initiating but not executing saccades to targets on the left compared to those on the right, as was the case in the first analysis. The initiation deficit, however, is disproportionately exaggerated when saccades are made leftward than rightward. This interaction between target side and direction of saccade is seen only in the initiation data and not in the duration or velocity data, again arguing against an impairment in saccadic execution.

The disproportionate increase in saccadic reaction time to contralesional targets with contralesionally-directed saccades is an important result; it not only helps in excluding the execution deficit as mediating the direction-specific saccadic problem but also allows us to localize more specifically the cause of the problem. The initiation deficit appears to arise partly from a perceptual problem in that targets on the left are always more poorly acquired than those on the right (and more poorly than the control subjects) and this is so even when a rightward saccade is made. However, the perceptual problem cannot account for the entire pattern of performance as targets on the left are acquired more poorly when the saccade to a target is leftward than rightward. In this condition, the target is located at a constant retinal distance and therefore should be detected equally well in both conditions. That there is a difference suggests that planning the saccade to the left is also impaired in these patients and implicates a problem that extends beyond perception. This result is important in so far as deficits in contralesional action have been largely attributed to patients with more anterior lesions, affecting frontal regions [15,25]. Instead, these findings implicate a role for parietal cortex in planning action in humans [39].

Having established the pattern of data, there are several issues that we need to address concerning the interpretation and possible artifacts before we relate the findings to more general issues. The first issue concerns the claim that the perceptual information is truly held constant when we manipulate the starting position of the eye. Although the position of the target was held constant with respect to the LED array and the midline of the head and trunk and retina, there are data to suggest that there is gain modulation of the salience of the target by virtue of the eye position or eye-in-head signal [70]. These findings come both from single neuron recording studies with animals [3,5] as well as from our own findings with neglect patients [10]. If it is indeed the case that the position of the orbit with respect to the head affects the perceptibility of the target, then the claim that perceptual position is absolutely identical in the EC and EL and ER cases might not be totally correct.

A further issue that warrants consideration is whether the pattern that we have observed (slower performance for left targets with leftward than rightward saccades) arises from a mechanical artifact. It is known that it is easier to move one's eyes back towards the midline or resting position than away from it. Because of the orbital elasticity and torques required by the extraocular muscles during movement, centripetal saccades (in) are easier than centrifugal (out) saccades [17,74]. Because leftwards saccades to left targets require an 'out' saccade and rightward saccades to left targets require an 'in' saccade, the pattern we see might be purely an artifact of the orbital mechanics. We think that this explanation is unlikely for two reasons. The first is that the differential ease of leftward than rightward saccades refers to the mechanism of execution rather than initiation and the deficit we observe is in initiation rather than in the movement per se. Additionally, if the mechanical difference constituted the explanation of the data, then one might also have expected to observe a similar result for targets on the right with rightward saccades for right targets ('out' saccades) being slower than leftward saccades for right targets ('in' saccades). In fact, we find no differences for right targets, suggesting that this mechanical account is likely not to be the total explanation.

Another important issue is with respect to what spatial coordinates 'left' and 'right' are defined as far as target position is concerned. This question bears critically on the issue of the co-ordinates or reference frame used to code information in space [4,8,22]. Unfortunately, we cannot easily differentiate the reference frames as left and right positions are coded with respect to the environmental angle (defined by the LED array with the central LED set to 0°) as well as with respect to the midline of the head and trunk as they are all aligned. The issue of spatial representation and reference frame is an important one and is the subject of ongoing investigation.

The final issue we need to examine before understanding the implications of the data has to do with whether the pattern we have observed is a function of neglect or of a lesion to parietal cortex. As is well known, there is not a one-to-one correspondence in general between neglect and parietal lesions; although neglect appears to occur most frequently and with greatest severity after parietal lesions, lesions to many other regions also give rise to neglect [13,46,60,80]. All of our patients demonstrated neglect. In addition, four of the five patients had clear lesions to parietal cortex with the fifth having a thalamic lesion which ostensibly de-afferents parietal cortex. Because of the confounding of neglect and lesion site, we cannot differentially attribute the eye movement pattern to one or the other source. To definitively address this issue and disambiguate the underlying mechanism, we would obviously need to have patients with neglect and other lesion sites or with parietal lesions and no neglect. This is yet to be done.

Having established that the problem appears to be more in the initiation or planning of the saccade than in the execution in our patients, we can begin to map these findings onto our knowledge of the saccadic system and to explore, in further detail, what mechanism gives rise to the deficit. As is well known, there are multiple brain regions involved in eye movements, including parietal cortex, the frontal eye fields (FEF) and the superior colliculus (SC) (see [17,76,77]). Saccades are initiated when omnipause neurons pause (opening a neural gate), allowing burst cells to fire and a saccade to be triggered. Burst cell activity codes the spatial metrics of the saccade (in higher centers such as the SC). The saccade is terminated when the omnipause neurons become active again (via a signal from fixation region in SC) holding the eye constant in its current position.

Where in this process might this initiation deficit arise? Because neither the FEF for SC is involved in these patients, the origin of the problem is almost certainly to be parietal and in the altered signal transmitted from parietal cortex to the other eye movement brain regions. Recent studies with monkeys have confirmed that the neuronal signal sent from parietal cortex to the superior colliculus carries both visual and saccade-related information. In a single unit recording study, Paré and Wurtz [64] antidromically activated neurons in lateral parietal cortex with single-pulse stimulation delivered to neurons in the intermediate layers of the SC. Many of the parietal neurons recorded exhibited sustained activity even when eye movements were not required and many of them also showed an increase in activity just before the onset of the saccade. One might easily translate this to the pattern we have seen in the patients here. Because of the unilateral lesion to parietal cortex, visual information about the contralateral target is likely to be compromised as is contralateral saccadic information. Because additional time is required to resolve this information (for activation to cross threshold, for example), additional time is required prior to or during transmission to SC. This manifests as slowing in initiation time. Because, in the patients with hemispatial neglect, the spatial metrics of the saccade are not differentially distorted for contralesional versus ipsilesional targets, it does not seem that the problem is one of holding the neural ‘gate’ open during the saccade. Rather, the problem appears to emerge during the transmission of information from parietal cortex to other eye movement regions of the brain.

The finding that patients with neglect who fail to attend to or process contralateral information have a mirror deficit in eye movements dovetails well with the growing functional imaging literature on the overlap between the attentional and eye movement systems (for example [24,63]). Additionally, a recent functional imaging study by Perry and Zeki [65] in which they used event-related functional MRI to differentiate shifts to the left and right has revealed a special role for the right supramarginal gyrus (part of the inferior parietal lobule) in saccadic processing and attention shifts. It is likely that this area which is probably damaged in our patients (or its function compromised by a remote lesion) is the source of the pattern we have observed.

Finally, of interest is that the interaction between target side and saccadic direction we have observed mirrors the finding of Mattingley and colleagues [39,57] in patients with posterior parietal lesions in a manual reaching task. This correspondence between the eye and hand movement data in patients with parietal lesions supports the view that the same underlying system or operating principles may be mediating both forms of action (see also [1,79]), despite the evidence to the contrary (normal subjects [54], patients [52] and single unit recording studies [33]). This pattern we have seen is also consistent with recent data from single-cell recordings in the posterior parietal cortex of awake, behaving monkeys, which demonstrate parietal involvement in the initial stages of motor planning. For example, Snyder and colleagues [79] found that 85% of posterior parietal neurons showed activity which was dependent on the type of movement (saccade or reach) being planned to a location in the cell's receptive field.

In sum, the findings we have obtained suggest that parietal cortex plays a specific role in directing action to different sides of space. Following a lesion to this area or to an area that impinges on it, planning an action to contralateral targets is impeded especially if the action itself is also contralesionally directed. These results highlight the role of parietal cortex in the domain of action and suggest that its function extends beyond one that is purely sensory in nature; rather it suggests that the role of parietal cortex might be more fruitfully thought of as operating at the interface of sensorimotor mediation [71].

## Acknowledgements

This work was supported by a grant from the National Institutes of Health to MB (MH54246). We thank John Sweeney for use of his lab for data collection and Ben McCurtain for help with the data analysis software. We also thank Jim Nelson and Thomas McKeeff for help with data collection, Sarah Shomstein for assistance with data analysis and Rob Kass for useful discussions. Finally, we thank Carl Olson for his comments on an early version of this paper and Richard Andersen and Larry Snyder for their helpful comments.
